# Does geographic spending variation exacerbate healthcare benefit inequality? A benefit incidence analysis for Indonesia

**DOI:** 10.1093/heapol/czab015

**Published:** 2021-06-02

**Authors:** Novat Pugo Sambodo, Eddy Van Doorslaer, Menno Pradhan, Robert Sparrow

**Affiliations:** Erasmus School of Health Policy and Management (ESHPM), Erasmus University Rotterdam, The Netherlands; Center for Health Financing Policy and Health Insurance Management, Faculty of Medicine, Public Health and Nursing, Universitas Gadjah Mada, Indonesia; Erasmus School of Health Policy and Management (ESHPM), Erasmus University Rotterdam, The Netherlands; Erasmus School of Economics (ESE), Erasmus University Rotterdam, The Netherlands; Vrije Universiteit Amsterdam, University of Amsterdam, and Tinbergen Institute, The Netherlands; Wageningen University and Erasmus University Rotterdam, The Netherlands

**Keywords:** Benefit incidence analysis, healthcare spending, social health insurance, universal health coverage, inequality, Indonesia

## Abstract

The Indonesian government has made some ambitious steps to achieve Universal Health Coverage through the newly formed National Health Insurance [*Jaminan Kesehatan Nasional* (JKN)], establishing a single-payer insurance agency and prospective provider payment mechanism. This study aims to assess the benefit incidence of healthcare funding in the JKN era, and its distribution by socio-economic status considering regional variation in unit costs. We evaluate whether the benefit incidence of funding is skewed towards urban and wealthier households. We also investigate whether standard benefit incidence analysis using national unit costs underestimates regional disparities in healthcare funding. Lastly, we examine whether the design of the JKN provider payment system exacerbates regional inequalities in healthcare funding and treatment intensity. The analysis relies on Indonesia’s annual National Socio-economic Survey (Susenas) and administrative data on JKN provider payments from 2015 to 2017, combined at district level for 466 districts. We find that the benefit incidence of healthcare expenditure favours the wealthier groups. We also observe substantial variation in hospital unit costs across regions in Indonesia. As a result, standard benefit incidence analysis (using national average unit transfers) underestimates the inequality due to regional disparities in healthcare supply and intensity of treatment. The JKN provider payment seems to favour relatively wealthier regions that harbour more advanced healthcare services. Urban dwellers and people living in Java and Bali also enjoy greater healthcare benefit incidence compared to rural areas and the other islands.

**KEY MESSAGES**

- Benefit Incidence Analysis using standard unit costs underestimates the magnitude of inequality in the benefit of healthcare spending.
- Unit costs for hospital services in Indonesia show substantial regional variation.
- Geographic variation in health service unit costs exacerbates inequality in the healthcare benefits in Indonesia.
- The Indonesian National Health Insurance maintained but did not increase initial disparities in treatment intensity and financing of healthcare.

## Introduction

Indonesia introduced the National Health Insurance System [*Jaminan Kesehatan Nasional* (JKN)] in 2014 to achieve universal health coverage by 2024. Mandated by Indonesian Law Number 40 in 2004 regarding the National Social Security System, the JKN consolidated existing mandatory social health insurance schemes (public servants, military, police and the formal private sector) and the subsidized insurance to the poor. In addition, informal sector workers, accounting for around 60% of the Indonesian labour force, were required to self-enrol. JKN is arguably one of the largest single-payer health insurance systems in the world (The Lancet, 2019). By October 2020, JKN had enrolled around 223.5 million members, representing ∼82% of the Indonesian population (DJSN, 2020a, b). JKN covers care from both public and private providers, including 22 971 out of 27 694 primary care providers (83%), 2487 out of 2925 hospitals (85%), as well as groups of pharmacists and medical laboratories (DJSN, 2020a, b; BPJS Kesehatan, 2018; Ministry of Health, 2020).

One of the main criticisms of the JKN design concerns its provider payment system that would be favouring municipalities and the better-off regions (Trisnantoro, 2019). With the introduction of prospective payments for secondary care, the compensation to healthcare providers increases with delivering more advance services, which are usually more abundant in urban hospitals and clinics. Primary healthcare payments under JKN are capitation based, determined by the number of enrolled members registered in the service catchment area, the available providers and the treatment intensity of service provision. As a result, better-equipped service providers are more likely to receive relatively larger provider payments, which may exacerbate regional disparities in healthcare treatment intensity and value.

Previous studies of the benefit incidence of healthcare spending in Indonesia have relied on socio-economic variation in healthcare utilization combined with constant national unit costs of healthcare (Lanjouw *et al.*, 2002; O’Donnell *et al.*, 2007). An important limitation of constant unit costs is that it assumes that the same type of healthcare service offers the same treatment intensity and value across the country. Utilization rates and national unit costs do not capture geographic disparities in healthcare service and intensity of treatment. For example, utilization of healthcare at an advanced hospital in the capital Jakarta will most likely be more intensive and involve more resources than the same type of service provided at a lower level hospital in a remote district, and thereby reflect a larger monetary value in offering the same public service. In this case, a benefit incidence analysis based on constant national unit costs will underestimate regional and socio-economic inequalities in who benefits from of healthcare spending in Indonesia.

Therefore, our study conducts a Benefit Incidence Analysis (BIA) of healthcare financing in Indonesia, using Indonesia’s JKN funding of healthcare providers to account for regional disparities in healthcare supply and intensity of treatment. We have three main objectives. First, we assess the benefit incidence of healthcare spending by region and socio-economic status and evaluate whether this spending is skewed towards urban and wealthier households. Second, we investigate whether standard BIA using constant national unit costs underestimates regional disparities in JKN funding to healthcare providers. Third, we examine whether the design of the JKN provider payment system exacerbates regional inequalities in healthcare supply and treatment intensity.

This article contributes to the BIA literature by using healthcare provider claims and capitation data of JKN, which provides accurate and detailed regional variation in unit costs for different types of healthcare services. The data are used to calculate unit costs at district level, reflecting the monetary value of health service offered in those districts as well as the regional variation in treatment intensity and supply of healthcare. By comparing these results to a standard BIA approach (with constant national unit costs), we can quantify the bias in the benefit incidence. A small number of studies use administrative data to capture regional differences in healthcare spending, for example, in the case of Australia (Ellis *et al.*, 2013) and Hungary (Bíró and Prinzba, 2020). Moreover, few studies distinguish regional variation in spending for hospital and primary care in low- and middle-income countries. For example, Anselmi *et al.* (2015) study differences in regional spending in Mozambique but limit their scope to outpatient care at primary and secondary facilities.

We also contribute to further understanding of the distributional implications of Indonesia’s JKN. Johar *et al.* (2018) use the National Socio-Economic Surveys from 2011 to 2016 to show that equity in access to healthcare improved after the introduction of JKN. Based on household panel data from the Indonesian Family Live Survey (IFLS) from 1993 to 2014/15, Mulyanto *et al.* (2019) find similar patterns for inpatient utilization but not for outpatient care in the first year of the JKN. Also using the IFLS data, for 2007 and 2014/15, Erlangga *et al.* (2019) show that JKN increased utilization of outpatient and inpatient care, but they question whether this reduces inequities in access, as the effects were larger for the self-enrolled group than for the subsidized poor. Health Policy Plus and TNP2K (2018) analyse JKN hospital expenditure and find that expenditure shares for the islands of Java, Bali and Sulawesi are disproportionately large relative to their population size, as is the expenditure share enjoyed by the rich. Our BIA analysis adds to these studies, as we assess JKN spending on both primary and secondary care, accounting for almost all of JKN disbursements. In addition, while inequity in access may have been declining over time, we demonstrate that ignoring regional variation in treatment intensity of care and the allocation rule underlying the provider payment system will underestimate the disparities in benefit incidence.

We assess the health benefit incidence by combining the National Socio-economic Survey (Susenas) and administrative data from the Health Insurance Agency (BPJS-*Kesehatan*). These two data sources cover a 3-year period from 2015 to 2017. The Susenas data provide information on per capita expenditure and healthcare utilization for various types of healthcare. In addition, BPJS-*Kesehatan* administrative data on provider payments allow us to construct *district specific unit costs* for these health services, aggregated into primary outpatient care, and secondary inpatient and outpatient care.

We find that the benefit incidence of healthcare funding is skewed towards the wealthier groups, and that using constant national unit costs underestimates the inequality in benefit incidence of healthcare spending for all types of care. However, we find no changes in the overall benefit incidence distribution during the first 3 years of the JKN, suggesting that its provider payment mechanism maintains geographic and socio-economic disparities but does not exacerbate these over time. Urban dwellers and people living on Java and Bali also enjoy a greater healthcare benefit compared to rural areas and the other islands.

The next section elaborates on the JKN financing system. Methods section sets out the BIA methods, and Data section describes the data. In Results and Discussion section, we present and discuss the results and Conclusion section concludes.

## JKN and healthcare financing in Indonesia

### Provider payment in the JKN era

A defining feature of the JKN reforms in Indonesia is the implementation of a single-payer healthcare system, by establishing the Social Security Agency in Health (*Badan Penyelenggara Jaminan Sosial-Kesehatan—*BPJS *Kesehatan*). As a single-payer for health insurance in Indonesia, BPJS-*Kesehatan* is responsible for the provider payments and collecting the premium contributions. The Indonesian government, through the Ministry of Health, sets the service standards and rules for the referral process. JKN members are not limited in seeking primary health care but need to obtain a referral to access higher level care. Primary care facilities (public and private) have a role as gate-keeper to regulate the flow to secondary hospital and tertiary specialized care.

Payment systems for secondary care are claims-based and regulated through Case-Based Groups (CBGs) of diagnosed-related groups called InaCBGs (Indonesian CBGs), which are calculated based on grouping diagnoses and procedures with similar clinical characteristics, resources and treatment costs. InaCBG tariffs are determined by the class of hospital (class A, B, C, or D), leading to relatively larger claims for more advanced hospitals. To encourage the involvement of private sector providers in JKN, InaCBG tariffs were increased by 3% for private inpatient and by 5% for private outpatient care (Agustina *et al.* 2019). The InaCBG tariffs also accommodate price differences across regions.^1^

Primary care providers registered with BPJS-*Kesehatan* receive JKN funding predominantly through a capitation scheme for outpatient service. In the capitation scheme, primary care providers receive a monthly upfront payment per JKN participant registered at the facility, irrespective of the actual services delivered. Capitation payments are meant to encourage independence and flexibility of primary care providers in managing their finances. Community health centres that meet the full requirements of BPJS-*Kesehatan* receive 6000 IDR (around 0.46 USD) per member per month.^2^ This capitation amount is reduced when these facilities fall short of the requirements. Private providers receive larger capitation amounts, ranging from 8000 (0.57 USD) to 10 000 IDR (0.71 USD), depending on their medical staff and service availability. Some services are not covered by capitation payments but are funded on a fee-for-service basis (referred to as non-capitation cases), such as antenatal care, deliveries and family planning services.

### JKN sources of payment

The JKN funding is pooled and distributed centrally by BPJS-*Kesehatan.* The funding comes from various sources. First, funding from the national government, coordinated by the Ministry of Finance and Ministry of Health, is earmarked for the subsidized insurance targeted to the indigent (37% of the JKN budget in 2016), and the premiums for civil servants, state-owned enterprise employees, and military and police (23%). Second, some provincial and district governments provide funding to cover premiums for informal sector workers that are required to self-enrol (5%). Third, voluntary registered informal sector workers contribute monthly premiums out-of-pocket (9%). Finally, the private formal sector has the responsibility to register their employees and share in the contribution of their JKN premiums (26%). Table 1 summarizes the shares of each source of contributions to the JKN.

**Table 1:** Distribution of JKN source of payment (%)

Type of membership	2014	2015	2016
Subsidized poor and indigent (national government budget) (A)	49.0	37.7	36.8
Civil servants, state-owned enterprise, military and police (national government budget) (C)	34.4	28.5	22.8
Self-enrolled subsidized by districts and provinces (local government budgets) (B)	3.3	4.5	5.4
Self-enrolled voluntary (individual premiums) (D)	4.6	8.9	8.5
Private sector (individual premiums) (E)	8.7	20.5	26.4
Total government share (A + B + C)	86.7	70.7	65
Total private share (D + E)	13.3	29.4	34.9

*Source*: Ahsan (2017).

In absolute amounts, total JKN financing almost doubled from 3.23 billion USD in 2014 to 6.26 billion USD in 2017.^3^ Hospital inpatient claims accounted for 3.67 billion USD or around 58.6% of total JKN funds disbursed in 2017, while 1.66 billion USD (or 26.4%) was allocated to hospital outpatient care. Combined, payments for hospital curative services amounted to 85% of the total JKN budget. Primary care services take a share of around 14% of the budget, even though outpatient primary care utilization accounts for around two-thirds of all JKN patient contacts.

### Benefit coverage

The JKN program covers a basic healthcare benefit package, including outpatient and inpatient care (starting from the appointed primary care, and up to secondary and tertiary care based on referral), maternal and child healthcare, dental health services (basic and advanced), advanced health services such as cancer therapies and haemodialysis, as well as health-related equipment with limited upper value or quantity, such as eyeglasses and hearing aids. Some healthcare is excluded, such as cosmetic treatment. Patients with JKN coverage are exempt from co-payments for medicine and medical items as long as the appropriate referral stages have been followed (Agustina *et al.*, 2019). In principle, co-payments are not allowed under JKN. In practice, out-of- pocket payments are still widely observed. This could be due to, for example, ex post upgrading of ward class, purchasing over-the-counter medicine outside treatment facilities and products that are not based on prescriptions, or traditional medicine.

The medical services provided under JKN are the same for all patients, but the class of ward may differ. Non-contributory subsidized JKN members are entitled to basic third-class hospital rooms for inpatient service. A self-enrolled or formal private sector JKN member may choose a class of hospital room (first, second and third class) that corresponds with their monthly minimum premium. It is also possible to take up private health insurance as a supplementary to JKN in order to cover extra benefits such as upgrades for hospital rooms, while upgrades can also be purchased out-of-pocket.

## Methods

Benefit Incidence Analysis (BIA) aims to evaluate the distribution of a public subsidy by socio- economic status within a population (e.g. Demery, 2000; O’Donnell *et al.*, 2008; McIntyre and Ataguba, 2011). We interpreted utilization of healthcare services as a transfer (or benefit) of public health spending (or subsidy) to an individual. BIA then described at an aggregate level how different population groups benefit from overall public health spending; for example, to what extent the benefits go disproportionately to the poor (e.g. due to government targeting) or to the wealthy (e.g. due to better access to public services).

### Average benefit incidence analysis with constant unit costs

To formalize the total healthcare financing benefit, we aggregated the transferred subsidy of all types of health services. This total subsidy for healthcare supplied under JKN was channelled through healthcare providers in the form of hospital claims (*T*^*cl*^) for inpatient and outpatient care, capitation funding (*T*^*cap*^) for primary outpatient care and non-capitation reimbursements (*T*^*non-cap*^) for primary inpatient care:

(1) $$T = {T^{cl}} + {T^{cap}} + {T^{non - cap}}.$$

To assess how this total subsidy was shared among socio-economic groups or regions, we need to consider who used healthcare. Using healthcare is valued at the unit cost of a healthcare service, ${{{T_i}} \over {{Q_i}}}$ defined as the average provider payment for reimbursing healthcare facilities for a delivered service *i* (i.e. outpatient or inpatient care at primary or secondary providers). Typically, standard BIA relies on the assumption that this unit cost is constant across the population. The total subsidy *S*_*ij*_ that was transferred to socio-economic group *j* for service *i* is then calculated by multiplying total utilization of the group, *Q*_*i*_, with the unit cost

(2) $${S_{ij}} = {Q_{ij}}{{{T_i}} \over {{Q_i}}}.$$

Aggregating the subsidy for all services *i* received by group *j* yields the total benefit incidence. Finally, dividing the total transfer *S*_*ij*_ by total JKN healthcare spending *T* expresses the total benefit incidence in terms of the shares of the transfer received by group *j:*

(3) $${S_j} = \mathop \sum \limits_i {{{Q_{ij}}} \over {{Q_i}}}\left[ {{{{T_i}} \over T}} \right].$$

### Average benefit incidence analysis with regional variation in unit costs

The availability of information on JKN spending by district allowed us to test whether the provider payment system affects the distribution of benefit incidence of public health spending. With the claims and capitation data, we can relax the assumption of constant unit costs and allow for variation by district. As the JKN claims were based on InaCBGs, they offer a realistic reflection of the variation in supply and the treatment composition of care offered in districts. We therefore assume that the district specific JKN unit costs are a good proxy for the regional variation in the implicit subsidy of healthcare utilization. The unit costs for service *i* in district *k* is then calculated by dividing the JKN transfer amount to the district *k* for that service $T_i^k$ by the number of units of care on which the JKN claims in district $k\left( {q_i^k} \right)$ are based, as measured in BPJS-*Kesehatan* administrative records. The average subsidy amount per unit of care used in quintile *j* for service *i* in district $k\left( {S_{ij}^k} \right)$ is obtained by multiplying this unit cost by the utilization of this group $\left( {Q_{ij}^k} \right)$ as measured in the Susenas survey:

(4) $$S_{ij}^k = Q_{ij}^k{{T_i^k} \over {q_i^k}}$$

The implicit assumption is that also non-JKN use of care in the district receives the same subsidy as JKN funded care. We can define spending in equation (4) for four different categories of services: hospital inpatient (HI), hospital outpatient (HO), primary outpatient (PO) and primary inpatient care.^4^ The relevant data on JKN spending came from the hospital claims for inpatient services $\left( {T_{HI}^k} \right)$ and hospital outpatient services ($\left( {T_{HO}^k} \right)$), and the primary care capitation payments ($\left( {T_{cap}^k} \right)$) to districts. We excluded primary care non-capitation claims from our calculations because this amount is relatively small, at around 1% of JKN spending (Pusat Pembiayaan dan Jaminan Kesehatan Kementerian Kesehatan, 2017). Total healthcare spending *T* thus reflects the summation of national hospital claims and capitation payments. The overall healthcare benefit for group *j* can then be expressed as

(5) $${S_j} = \mathop \sum \limits_k Q_{HIj}^k{{T_{HI}^k} \over {q_{HI}^k}} + Q_{HOj}^k{{T_{HO}^k} \over {q_{HO}^k}} + Q_{POj}^k{{T_{cap}^k} \over {q_{PO}^k}}.$$

The proportion of the benefit transferred to socio-economic group *j* is then written as

(6) $${S_j} = {S_j}/\mathop \sum \limits_j {S_j}.$$

### Concentration curve and concentration index

We used concentration curves to illustrate the relative inequality of healthcare benefit. Concentration curves describe the benefit incidence of healthcare by plotting the cumulative proportion of healthcare use against the cumulative proportion of the population ranked by per capita household expenditure per adult equivalent (O’Donnell *et al.*, 2008). If the healthcare funds are distributed pro-poor then the concentration curve should lie above the 45° equity line, whereas it would fall below the equity line in case of a pro-rich distribution.

The inequality implied by a concentration curve can be expressed in terms of a concentration index, which reflects twice the area between the concentration curve and the diagonal (Wagstaff and van Doorslaer, 2000). The concentration index can be calculated as

(6) $$C = {1 \over n}\mathop \sum \limits_{i = 1}^n {{{h_i}} \over {\bar h}}\left( {2{R_i} - 1} \right),$$

where *n* is the sample size, *h*_*i*_ is an individual’s healthcare benefit in monetary terms with mean $\bar h$, and *R*_*i*_ is the fractional rank of individual’s in the distribution of per capita expenditure (with *i* = 1 for the poorest and *i* = *n* for the richest). The value of the concentration index is negative for a pro-poor distribution of the JKN funding and positive for a pro-rich distribution, while an equal distribution yields a concentration index of zero. Dominance of distributions can be verified (and tested) by checking whether a concentration curve lies everywhere above another curve or above the Lorenz curve measuring household income/expenditure inequality (O’Donnell *et al.*, 2008).

### Data

We combined the National Socio-economic Household Survey (Susenas) for 2015, 2016 and 2017 with administrative data from BPJS-*Kesehatan* over the same time period at district level. The Susenas data are representative at national and district level and provide information on utilization of primary and secondary healthcare services, and per capita expenditure. The sample size of Susenas is 1 097 719 individuals in 2015, 1 109 749 in 2016 and 1 132 749 in 2017. We used the Susenas sampling weights to ensure that the micro data is representative at the district and national level. BPJS-*Kesehatan* data provide JKN hospital claims data and capitation payments for 466 of Indonesia’s 514 districts, and also records inpatient and outpatient contacts/consultations of JKN registered individuals per district.

To implement the BIA with our combined data, we proceeded in several steps. First, we calculated the unit costs based on the BPJS-*Kesehatan* data. The unit costs of each health service and for each district are calculated by dividing the total sum of district JKN claims (from all JKN-registered hospitals) by the total outpatient consultations or inpatient contacts in a district. Unfortunately, the utilization of JKN members of outpatient primary care ($\left( {q_{PO}^k} \right)$) is not in the JKN administrative data as the payments are made on a capitation basis. We therefore estimated this using Susenas data by multiplying the total utilization for outpatient primary care services in district *k* by the fraction of the population in the district *k* that is a JKN member.

We can interpret the unit cost or unit transfer as the monetary value of a treatment and assume that it varies with the intensity of care provision in districts. As our analysis relies on JKN disbursements as a proxy for healthcare financing disparities across regions, we also assume that the variation in district mean JKN spending appropriately captures the actual variation in healthcare benefits obtained for *all* district inhabitants, including both JKN members and non-members.^5^ This assumption is valid if unit costs are supply driven, and JKN and non-JKN members use similar services.

Second, we use the Susenas household survey data to calculate the distribution of healthcare utilization by socio-economic group per district (*Q*^*k*^_*ij*_). The household survey offers socio-economic characteristics of the district populations that the BPJS-*Kesehatan* does not include. We measure contact rates of each type of healthcare in Susenas, which records individual hospital or primary outpatient care in the month before the survey (around March for each year). We define primary care facilities as community health centres (*Puskesmas*) and their local subsidiaries, polyclinics and GP practices, while exclude traditional practices. The inpatient care recall period is the year preceding the survey. Because the district claims data is on an annual basis, we annualized outpatient use.

To evaluate the cost of health services distributed across rich and poor, we ranked individuals based on the national distribution of per capita expenditure and define quintiles (where quintile 1 is the poorest group and quintile 5 the wealthiest). Per capita expenditure as well as the unit costs were also adjusted for regional price differences using a provincial consumer price index (taking Jakarta province in 2014 as base year).

## Results and discussion

### How do unit costs vary by district?

**Figure 1:**
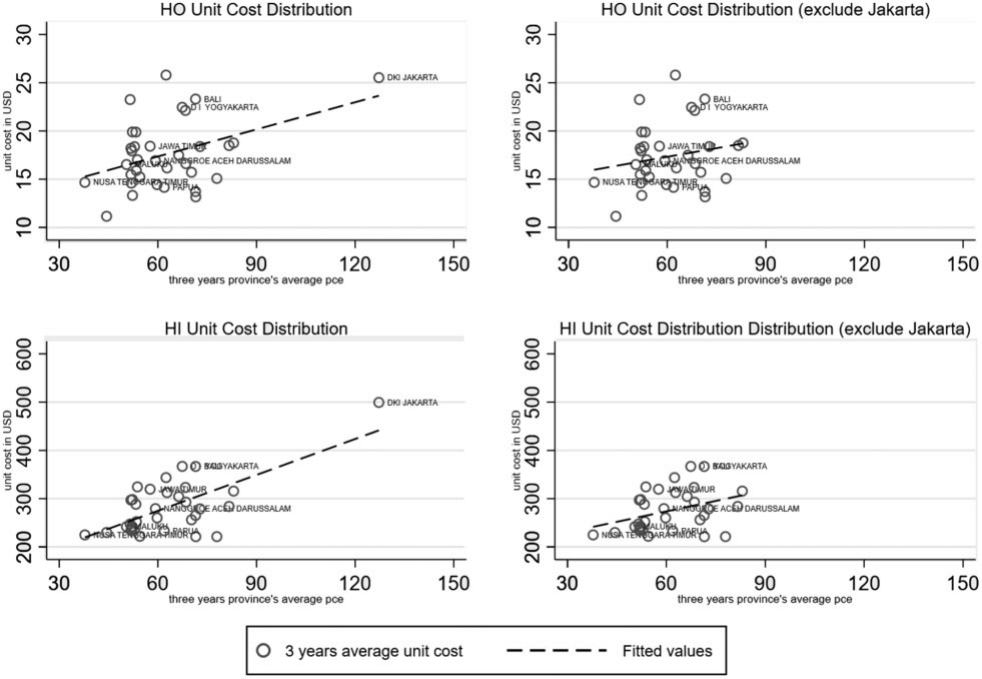
Association between mean unit cost and mean household expenditure, by province, for outpatient and inpatient hospital care. *Note*: HO, hospital outpatient; HI, hospital inpatient care. The Y-axis shows 3 years average of province specific unit costs derived from the BPJS-*Kesehatan* records on inpatient and outpatient claims and cases per district. The X-axis shows 3 years province averages of per capita expenditure derived from Susenas 2015 to 2017. The dash line represents a linear regression line. *R*^2^ include Jakarta HO (0.1742) and HI (0.4559). *R*^2^ exclude Jakarta HO (0.0456) and HI (0.1296).

The procedure described in the previous section generates an estimate of the regional variation in the unit cost of different healthcare services across districts. We find a positive correlation between average per capita household expenditure and hospital unit costs (Figure 1). The relatively expensive care provided in Jakarta drives much of this association, with a unit cost of 26.50 USD per hospital outpatient contact (about 50% higher than the national average of 17.50 USD) and 530 USD per inpatient care contact (i.e. about 80% higher than the national average of 291.80 USD). Nevertheless, a positive correlation remains visible when we exclude Jakarta from the scatterplot.

The variation in regional hospital unit costs in Figure 1 may be related to the availability of more advanced type hospitals (Hospital class A or B) or tertiary healthcare providers. A district or city with a class A (tertiary care) hospital will receive greater JKN disbursements than those without because they can offer a wider variety of medical services. These more advanced hospitals are not equally distributed across the country. Trisnantoro (2019) reports that two-thirds of the 61 class A hospitals in Indonesia are located in Java Island and 16 of these are in Jakarta. Our approach attributes unit costs to the place of (hospital) delivery, not the place of residence of the user. So if a resident of another district than Jakarta receives (tertiary) hospital care in Jakarta, the benefit is attributed to Jakarta residents, not the non-Jakarta residents receiving it.

For primary care unit costs, we use annual capitation payments at district level over the period 2015–17 and Susenas primary outpatient service utilization. We find that district primary care unit costs do not vary much across districts, with the total capitation payments more proportional to the respective population sizes (see Supplementary Figure A4).

### Socio-economic distribution of health benefits after JKN

Table 2 provides an overview of the socio-economic distribution of the utilization rate, standard benefit incidence shares, district-weighted unit costs and weighted benefit incidence shares for all three types of healthcare services. We divide the population into five quintiles based on per capita household expenditure in each year.

**Table 2.** Distribution of healthcare utilization share across socio-economic quintiles (2015–17)

Hospital Inpatient						Hospital outpatient			Primary care outpatient			
	Contact per 100 individuals in one year	Share (%)	Unit Cost (USD)	Benefit Share % (district unit costs)	Contact per 100 individuals in one year	Share (%)	Unit Cost (USD)	Benefit Share % (district unit costs)	Contact per 100 individuals in one year	Share (%)	Unit Cost (USD)	Benefit Share % (district unit costs)
	1	2	3	4	5	6	7	8	9	10	11	12
2015												
Poorest	1.57	11.06	236.60	9.77	11.81	9.14	14.15	8.09	169.53	19.14	3.27	17.81
2nd quintile	1.94	13.44	243.00	12.23	15.74	12.32	14.76	11.46	181.69	20.53	3.44	19.80
3rd quintile	2.55	17.49	250.07	16.55	20.88	15.91	15.01	14.93	184.09	20.98	3.53	20.87
4th quintile	3.42	23.08	261.95	23.03	31.02	23.01	15.74	23.01	182.66	20.80	3.65	21.39
Richest	5.10	34.92	276.70	38.42	52.34	39.62	16.75	42.50	160.16	18.55	3.85	20.14
Mean	2.79	100	248.99	100.00	24.92	100	15.04	100.00	175.99	100	3.45	100.00
2016												
Poorest	1.52	10.31	223.54	9.24	12.17	9.46	14.02	8.42	156.30	18.92	3.46	17.60
2nd quintile	2.03	13.87	232.14	13.03	16.47	12.79	14.85	12.02	170.96	20.79	3.56	20.08
3rd quintile	2.52	17.29	233.66	16.32	20.13	15.72	14.98	15.13	171.26	20.77	3.67	20.47
4th quintile	3.56	24.41	243.49	24.29	28.86	22.70	15.25	22.41	175.59	21.27	3.76	21.82
Richest	4.98	34.12	256.08	37.12	50.31	39.33	16.09	42.01	150.72	18.26	4.03	20.02
Mean	2.93	100	235.13	100.00	25.64	100	14.91	100.00	164.89	100	3.60	100.00
2017												
Poorest	1.72	10.81	222.76	9.58	9.58	9.16	15.17	8.45	130.09	19.06	3.89	17.41
2nd quintile	2.34	14.70	234.03	13.84	13.48	12.76	15.45	12.16	138.32	20.08	4.01	19.10
3rd quintile	2.76	17.57	237.93	16.77	16.33	15.60	15.63	15.00	142.31	20.80	4.14	20.68
4th quintile	3.59	23.23	240.19	22.86	22.38	21.94	15.92	21.62	146.00	21.44	4.26	21.97
Richest	5.41	33.69	248.28	36.95	42.45	40.54	16.25	42.77	131.47	18.62	4.22	20.85
Mean	3.28	100	236.75	100.00	21.90	100	15.68	100.00	137.74	100	4.08	100.00

*Source*: Authors’ calculation based on BPJS-*Kesehatan* claims data and Susenas 2015–17.

*Notes*: Distribution of socio-economic quintiles is based on per capita expenditure of each year (regional CPI adjusted).

For the district-weighted hospital unit costs we find that the gap between the richest and poorest quintiles persists, although it does decline over time. Table 2 (column 3) shows that for the richest quintile the hospital inpatient unit costs in 2015 are 40 USD (17%) higher than for the poorest quintile. This difference declined to 26 USD (11%) in 2017. The unit costs for hospital outpatient care and primary care are smaller in nominal terms but show a similar gradient and trend (Table 2, column 7 and 11).

The utilization rates (per 100 individuals) for hospital inpatient, hospital outpatient and primary outpatient care in the past year are reported in columns 1, 5 and 9. Hospital utilization is highly skewed towards the wealthier groups. The hospital inpatient contact rate for the fifth quintile is almost three times that of the poorest quintile, and the hospital outpatient contact rate is more than four times larger. In contrast, outpatient contact rates at primary providers show a nearly equal distribution, with slightly higher rates for the middle quintiles.

The patterns in utilization are also reflected in the benefit incidence results. The benefit shares based on a standard BIA calculation with constant unit costs, as in equation (2), are presented for each type of healthcare service in columns 2, 6 and 10 of Table 2. The benefit shares based on district specific unit costs are presented in columns 4, 8 and 12. The standard benefit incidence share of hospital care for the wealthiest quintile increased over time, reaching 34% for inpatient care and 41% for outpatient care in 2017; whereas the shares for the poorest decline over time, to respectively 11 and 9%. Again, the standard benefit shares of primary care exhibit a much more equal distribution than hospital care.

When we allow for variation in the district unit costs, following equation (4), we see the discrepancy in benefit incidence of hospital care increases. For the wealthiest group the benefit share in 2017 increases to 37% for inpatient care, and to 43% for outpatient care. For the poorest the shares decrease even further, to 10 and 8%, respectively. A similar effect is observed for primary care. When we weigh the rather evenly distributed contact rates with the gradient in district specific unit costs, the resulting benefit incidence shares also turn pro-rich. The richest quintile now accounts for more than a 20% of the primary care spending, and the poorest quintile for 17% in 2017. This rise for the wealthier groups can partly be explained by JKN’s gatekeeping mechanism, where referrals from community health centres are required for higher level care to be covered. According to Johar *et al.* (2018), there is an increasing use of GPs and primary health centres. Before JKN the better-off could directly consult a specialist or hospital without referral from a GP. But after the implementation of JKN and its gatekeeping mechanism, the requirement to obtain a referral from a primary care facility was more widely enforced.

### National unit cost vs district-specific unit cost

**Figure 2:**
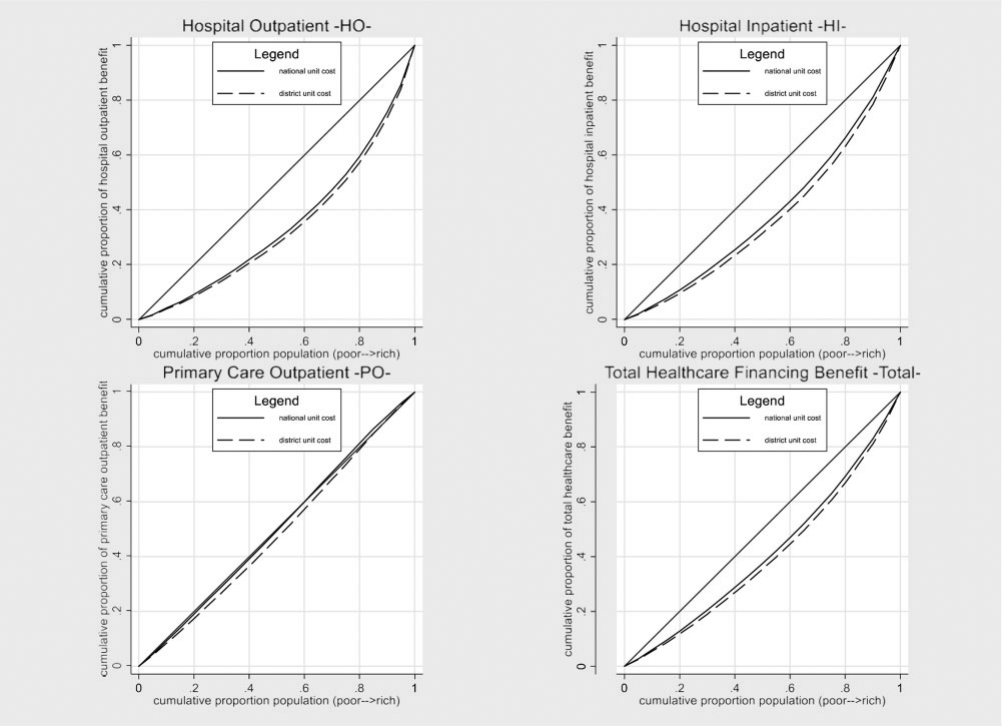
Comparison of concentration curves with national unit cost and district-specific unit cost, for hospital (outpatient and inpatient) and primary care outpatient benefit (2017). *Note*: HO: Hospital Outpatient; HI: Hospital Inpatient; PO: Primary Care Outpatient. The y-axis plots the cumulative density of a healthcare benefit incidence for individuals ranked by per capita expenditure in 2017.

The distributions of healthcare financing benefit incidence based on constant national average unit costs and district-specific unit costs are compared for 2017 in Figure 2 by means of concentration curves. We find that disparities in unit costs among districts generate a more pro-rich distribution. For all types of healthcare, the concentration curves for district-specific unit costs are dominated by the curves for constant unit costs, as the latter lie closer to the diagonal across the income distribution indicating a more equal benefit incidence. The associated concentration indices therefore take a positive value and are larger for the district-specific unit cost-benefit incidence, with the difference statistically significant at a 1% level for all types of care (significance tests of differences in the concentration indices are reported in Supplementary Table A2).

Finally, the concentration curves for the benefit incidence of total healthcare funding, following equation (5), also show a clear pro-rich distribution (bottom-right graph in Figure 2). The concentration index is 0.178 when based on constant unit costs, but increases to 0.211 when we allow the unit costs to vary by district. These results suggest that provider payments of the JKN favour the wealthier groups and regions. Conversely, ignoring this regional variation in provider payments that is driven by initial disparities in healthcare supply, will underestimate the inequality in benefit incidence of healthcare spending. However, the concentration indices do not change much over time and these differences are not statistically significant, irrespective of whether we allow for variation in unit costs (the concentration index increases from 0.210 to 0.211 between 2015 and 2017, using district unit costs; detailed tests are reported in Supplementary Table A3). This indicates that the regional inequality in JKN provider payments does not exacerbate the inequality in treatment intensity and supply of healthcare (and thereby the benefit incidence) over time.

### Healthcare financing benefit distribution based on geographic location

While we do not observe increasing inequities in benefit incidence over time as JKN was introduced, we do see a slight increase in disparities between and within regions. The urban share in healthcare expenditure was already larger than its 56% population share, and it increased very slightly from 64 to 66% between 2015 and 2017. The country’s economically most developed islands Java and Bali represent around 57% of the national population yet benefit from around 67% of overall healthcare spending. This disparity is largely due to expenditures on secondary care. The benefit incidence of outpatient primary care remains equally distributed, although the urban share is growing slowly, from 53 to 56% (results reported in the Supplementary Table A4). The rural share for the hospital inpatient benefit decreases slightly from 33 to 31% (Supplementary Table A4).

To assess changes in benefit incidence within regions, we compute concentration indices for the main island groups and for the municipalities and rural districts. For the municipalities and the islands Java and Bali, we see no statistically significant changes to the distribution of healthcare expenditure. However, for regions that were initially less endowed with healthcare supply, such as rural districts and the eastern islands (Maluku, Papua, NTT and NTB), we see a statistically significant increase in their concentration index value, suggesting an increased socio-economic inequality in the benefit incidence (results reported in Supplementary Table A5).

**Figure 3:**
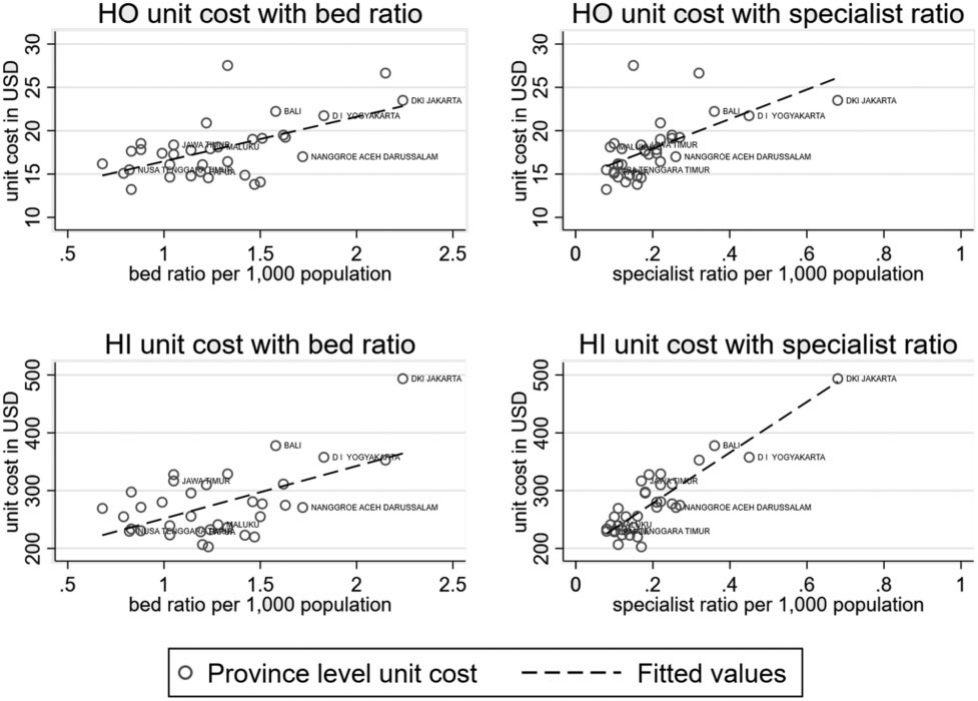
Hospital care unit cost and healthcare facility availability at province-level. (2017) *Source*: BPJS-*Kesehatan* claims data for 2017 and *Pusat Data dan Informasi Kementerian Kesehatan* (2018). *Notes*: HO, hospital outpatient; HI, hospital inpatient; PO, primary care outpatient.

The disproportionate share going to the more developed regions can be explained partly by variation in unit costs. Figure 3 plots the average hospital care unit costs at province level against the average bed ratios and specialist ratios per 1000 population. The scatterplots show positive correlations between hospital care unit costs and the availability of beds and specialists. This positive association reflects the fact that the JKN provider payment design favours regions with relatively abundant healthcare supply, thereby widening the gap in healthcare funding between regions.

### Limitations

One key limitation of the Susenas data is that the *number* of outpatient visits is reported only in 2017. For 2015 and 2016, the survey only records *whether* a respondent obtained outpatient care and at what facility. For inpatient care, on the other hand, the Susenas records the annual number of inpatient days for all years. For consistency we therefore use the contact rate as a proxy for utilization, to calculate the benefit shares and the total spending benefit. Using the number of outpatient visits and inpatient days would be preferable. In order to check the sensitivity of our results to this simplification, we replicate the analysis with outpatient visits and inpatient days for the year in which the number of outpatient visits is available. The results are provided in Supplementary Table A6. Two key observations emerge: (1) the gradient is slightly more pro-rich when we use utilization rates, but (2) the BIA results with constant and district unit costs are very similar. This confirms that our results are not affected by approximating utilization with the utilization fraction. We present these results in our article, as we prefer a consistent approach for all years and types of care in order to calculate the overall benefit share.

We also note that we do not assess out-of-pocket payments in this study, despite the fact that these still commonly occur in Indonesia. Our analysis focusses on the implicit public subsidy transfer of health care utilization and how this is distributed over the population. Out-of-pocket payments therefore fall beyond the scope of our article.

Another caveat relates to the portability principle of JKN, which enables a patient to move to another hospital in a different district, province or island in case of a medical necessity. This implies that a district that receives a JKN disbursement is not necessarily the residence of the patient that received the treatment. Given the available data, we cannot adjust for cross-district-border healthcare utilization. However, a recent study of inter-province mobility based on the BPJS-*Kesehatan* claims data from 2015 to 2016 finds that inter-province patient movement is negligible compared to total JKN funding.^6^

Finally, we may still underestimate the inequality in the distribution of healthcare benefits as we are only using provider payments at district level and do not include geographic variation in unit cost of higher class hospital services (tertiary care), level of severity, and case-mix-groups of disease prevalence. For example, treatment for more costly diseases (such as cancer or cardiovascular disease) is likely to be relatively higher in wealthier urban areas and correlated with knowledge, health awareness and access to care.

## Conclusion

Our research findings show that the benefits of healthcare spending since the introduction of Indonesia’s JKN program are distributed disproportionately favouring the wealthier population groups, as well as urban areas and islands Java and Bali. We also find substantial variation in healthcare unit costs across districts, because regions with well-equipped health facilities are associated with relatively higher unit transfers for healthcare services.

This variation in unit costs implies that BIA using national average unit costs will underestimate the disparities in healthcare funding. Previous studies that have analysed healthcare utilization under JKN (and ignoring regional differences in unit transfers) are therefore likely to overestimate the extent to which JKN has reduced inequality in healthcare delivery.

A second implication of our BIA results is that we can interpret the difference in benefit incidence based on constant and varying unit costs as the bias inherent in JKN’s provider payment mechanism: if the claims and capitation data were not biased towards wealthy regions and population groups, then any unequal distribution should be due to utilization patterns alone and the choice of unit costs should not matter. Nevertheless, we do not find statically significant changes to the concentration indices over time post-JKN, suggesting that JKN’s provider payment system maintained initial disparities in treatment intensity and funding of healthcare between 2015 and 2017, but did not exacerbate these as some had feared (Trisnantoro, 2019).

Two policy priorities emerge from our findings. First, to reduce inequities in healthcare funding across regions and population groups, the existing prospective payment mechanism would need to be modified using an affirmative or targeted design to promote a higher value of care in regions with less-developed healthcare facilities. One possibility here to be considered is to adjust the InaCBG tariffs depending on supply readiness gaps. This might make health infrastructure investment more attractive in these areas. A similar instrument is already in use for primary care in the *Dana Kapitasi Khusus* policy that creates a higher capitation funding for primary care in remote districts (Ministry of Health, 2016).

Second, the national and local governments could directly invest in the supply of healthcare facilities and staff in rural areas and districts outside Java and Bali. This is not an easy task, as witnessed by the same problem arising in many other countries. Especially difficult is attracting doctors and other medical personnel to work in remote places. Some inspiration can be obtained from the results of financial and non-financial incentives deployed in Thailand for medical school graduates to work in rural and remote areas (Wibulpolprasert and Pengpaibon, 2003). Similar policy suggestions have been provided by Chomitz *et al.* (1999). They concluded that offering specialist training may be a sufficient incentive to make doctors from Java willing to serve in remote areas, but that it is an expensive and potentially inefficient policy since specialist practice and rural public health management require different skills and attitudes. They claim that moderately (but not extremely) remote areas can also attract additional staff using modest cash incentives. They find that especially doctors originating from the Outer Islands are far more willing to serve in remote areas than their counterparts from Java. So, it may be worthwhile increasing the representation of Outer Island students in medical schools (perhaps through scholarships and assistance in pre-university preparation).

## Funding

Novat Pugo Sambodo gratefully acknowledges financial support from the Indonesian Endowment Fund for Education (LPDP).

## Supplementary data

Supplementary data are available at *Health Policy and Planning* online.

*Conflict of interest statement*. None declared.

*Ethical approval:* Ethical approval is not required as this research did not directly involve human-subjects and face-to-face interactions. The research is based on anonymized secondary data from Statistics Indonesia and administrative data from Indonesia’s Health Insurance Agency.

## Supplementary Material

czab015_Supp

## Footnotes

1 Based on the consumer price index, districts are classified into five groups. For each group a price correction factor is applied. The InaCBG tariffs therefore accommodate price differences between the five groups but ignore the variation within the groups or within districts.

2 One USD equals about 14 000 IDR (February 2020).

3 Based on BPJS-*Kesehatan* data. See Average benefit incidence analysis with regional variation in unit costs section for more details.

4 About 36% of total community health centres in Indonesia provide inpatient services, although the number of beds per facility is limited.

5 By December 2017, around 73% of the total population was enrolled into JKN and almost 80% of all hospitals in Indonesia had joined JKN in 2016. For primary care, all community health centres joined JKN.

6 Center for Health Policy and Management, Gadjah Mada University (PKMK UGM). Study details can be accessed (in Bahasa Indonesia) at: https://kebijakankesehatanindonesia.net/datakesehatan/file/Portabilitas-peserta-JKN.html.
