# Supplementary material for: Does geographic spending variation exacerbate healthcare benefit inequality? A benefit incidence analysis for Indonesia
Source: Health Policy Plan. 2021 Jun 2;36(7):1129–39. doi: 10.1093/heapol/czab015 (PMC8359753; doi:10.1093/heapol/czab015)
Supplement: czab015_Supp [file czab015_supp.zip › Supplementary_appendix_final.pdf]

<u>Supplementary Appendix [not for publication, but to be made available online]</u>

Table A1. Summary statistics of linked data between JKN hospital claim/capitation fund and Susenas (2015-2017)

Name of Variable	# observations	Mean	SD	Min	Max
**Individual Level Data (Susenas)**					
Average Per Capita Expenditure per month (USD) in Susenas	3,164,933	70.887	68.646	6.34	4,946.15
HH member using outpatient service in the last month (Yes=1; No=0)	3,164,933	0.149	0.356	0	1
Outpatient service in hospital in the last month (Yes=1; No=0)	3,164,933	0.020	0.139	0	1
Outpatient service in primary care in the last month (Yes=1; No=0)	3,164,933	0.128	0.334	0	1
HH member using inpatient service in the last year (Yes=1; No=0)	3,164,933	0.028	0.165	0	1
Number of inpatient days	3,164,933	0.169	1.654	0	364
Inpatient care in hospital in the last year (Yes=1; No=0)	3,164,933	0.030	0.170	0	1
inpatient care in primary care in the last year (Yes=1; No=0)	3,164,933	0.008	0.089	0	1
Living in urban are (Yes=1; No=0)	3,164,933	0.445	0.497	0	1
Municipality=1; District=0	3,160,271	0.208	0.406	0	1
Java-Bali=1; Non Java-Bali=0	3,164,933	0.336	0.472	0	1
**District Level Data (BPJS-Kesehatan)**					
Unit Cost for Hospital Inpatient visit per District (BPJS-Kesehatan data) (USD)	1,402	234.32	68.75	82.39	764.41
Unit cost for Hospital Outpatient visit per District (BPJS-Kesehatan data) (USD)	1,400	14.91	4.04	0.02	37.46
Unit cost for Primary Care Outpatient visit per District (BPJS-Kesehatan data + Susenas) (USD)	1,405	4.61	4.69	0.66	129.50

Table A2. Concentration index comparison using national unit cost vs. district-specific unit cost (2017)

Variable	DUC			NUC			DUC-NUC	
	CI	SE	p-value	CI	SE	p-value	CI	p-value
	1	2	3	4	5	6	7	8
Hospital Outpatient (HO) Benefit	0.335	0.007	0.000	0.307	0.007	0.000	0.028	0.000
Hospital Inpatient (HI) Benefit	0.269	0.005	0.000	0.229	0.005	0.000	0.040	0.000
Primary Care Outpatient (PO) Benefit	0.039	0.002	0.000	0.001	0.002	0.526	0.038	0.000
Total Healthcare Financing Benefit	0.211	0.003	0.000	0.178	0.003	0.000	0.033	0.000

Source: Authors' analysis based on Susenas 2017 and BPJS-*Kesehatan* administrative data.

Table A3. Concentration Index of healthcare financing benefit distribution (2015-2017)

Variable	2015 (SE) [p-value]	2016 (SE) [p-value]	2017 (SE) [p-value]	Diff:2016-2015 (SE) [p-value]	Diff:2017-2016 (SE) [p-value]	Diff:2017-2015 (SE) [p-value]
Using National Unit Cost (NUC)						
Hospital Outpatient Benefit	0.305	0.294	0.307	-0.012	0.014	0.002
	0.006	0.006	0.007	0.008	0.009	0.009
	0.000	0.000	0.000	0.166	0.125	0.824
Hospital Inpatient Benefit	0.243	0.243	0.229	0.000	-0.014	-0.014
	0.005	0.005	0.005	0.007	0.007	0.007
	0.000	0.000	0.000	0.932	0.028	0.038
Primary Care Outpatient Benefit	-0.004	-0.003	0.001	0.001	0.005	0.006
	0.002	0.002	0.002	0.003	0.003	0.003
	0.019	0.074	0.526	0.765	0.097	0.045
Total Healthcare Financing Benefit	0.209	0.210	0.213	0.001	0.003	0.004
	0.004	0.003	0.004	0.005	0.005	0.005
	0.000	0.000	0.000	0.889	0.523	0.438
Using District Unit Cost (DUC)						
Hospital Outpatient Benefit	0.343	0.328	0.335	-0.015	0.007	-0.008
	0.007	0.007	0.007	0.009	0.010	0.010
	0.000	0.000	0.000	0.119	0.480	0.426
Hospital Inpatient Benefit	0.288	0.281	0.269	-0.007	-0.012	-0.019
	0.005	0.005	0.005	0.008	0.008	0.008
	0.000	0.000	0.000	0.403	0.115	0.017
Primary Care Outpatient Benefit	0.025	0.027	0.039	0.001	0.013	0.014
	0.002	0.002	0.002	0.003	0.003	0.003
	0.000	0.000	0.000	0.650	0.000	0.000
Total Healthcare Financing Benefit	0.210	0.206	0.211	-0.005	0.005	0.001
	0.003	0.003	0.003	0.005	0.005	0.005
	0.000	0.000	0.000	0.317	0.261	0.908

Source: Authors’ analysis based on Susenas 2015-2017 and BPJS-*Kesehatan* administrative data..

Table A4. Healthcare financing benefit share based on geographic location (2015-2017)

Geographic Location		H0			HI			PO			Total		
		2015	2016	2017	2015	2016	2017	2015	2016	2017	2015	2016	2017
Rural vs Urban													
Share Benefit (%)	Rural	26.3	28.6	26.3	32.8	32.6	30.9	47.1	45.2	44.4	36.1	35.9	34.2
	Urban	73.7	71.4	73.7	67.2	67.4	69.1	52.9	54.8	55.6	63.9	64.1	65.8
Average odds	Rural	0.54	0.60	0.57	0.67	0.68	0.67	0.97	0.95	0.96	0.74	0.75	0.74
	Urban	1.44	1.37	1.37	1.31	1.29	1.29	1.03	1.05	1.03	1.25	1.23	1.22
Non Java-Bali Vs Java-Bali													
Share Benefit (%)	Non Java-Bali	31.0	31.9	30.6	32.3	32.8	32.2	35.7	36.4	37.5	33.1	33.8	33.5
	Java-Bali	69.0	68.1	69.4	67.7	67.2	67.8	64.3	63.6	62.5	66.9	66.2	66.5
Average odds	Non Java-Bali	0.76	0.79	0.75	0.80	0.81	0.79	0.88	0.90	0.92	0.82	0.83	0.82
	Java-Bali	1.16	1.15	1.17	1.14	1.13	1.15	1.08	1.07	1.06	1.13	1.11	1.12
Other Islands													
Share Benefit (%)	Sumatera	17.7	18.5	17.3	16.9	16.7	16.8	19.9	19.8	19.7	18.09	18.20	17.84
	NTB and NTT	2.4	2.0	2.3	2.5	2.3	2.3	3.4	3.0	3.5	2.72	2.46	2.67
	Kalimantan	4.4	4.6	4.3	5.0	5.5	5.0	5.0	5.1	5.7	4.87	5.15	5.06
	Sulawesi	5.0	5.2	5.1	6.5	6.9	6.8	5.0	6.4	6.3	5.65	6.33	6.31
	Maluku and Papua	1.5	1.6	1.6	1.3	1.4	1.3	2.4	2.1	2.3	1.76	1.68	1.64
Average odds	Sumatera	0.82	0.85	0.78	0.79	0.77	0.76	0.92	0.91	0.89	0.84	0.83	0.81
	NTB and NTT	0.64	0.52	0.60	0.66	0.61	0.60	0.89	0.79	0.91	0.73	0.65	0.69
	Kalimantan	0.75	0.77	0.68	0.86	0.93	0.79	0.85	0.86	0.91	0.83	0.87	0.81
	Sulawesi	17.7	18.5	17.3	16.9	16.7	16.8	19.9	19.8	19.7	18.09	18.20	17.84
	Maluku and Papua	2.4	2.0	2.3	2.5	2.3	2.3	3.4	3.0	3.5	2.72	2.46	2.67

Note: We use district unit costs to produce healthcare benefit shares. The average odds are a result of dividing shares of healthcare financing benefit received in a group by the share of its population to total population. We use population estimates from Susenas.

Table A5. Total healthcare financing benefit concentration index disparities based on geographical location (2015-2017)

Variable	2015<br>(SE)<br>[p-value]	2017<br>(SE)<br>[p-value]	Diff:2017-2015<br>(SE)<br>[p-value]
Using District Unit Cost (DUC)			
Urban	0.251	0.254	0.003
	0.006	0.006	0.009
	0.000	0.000	0.753
Rural	0.190	0.200	0.009
	0.005	0.005	0.007
	0.000	0.000	0.176
Kota (Municipalities)	0.152	0.145	-0.007
	0.008	0.007	0.011
	0.000	0.000	0.513
Kabupaten (District)	0.206	0.225	0.018
	0.004	0.005	0.006
	0.000	0.000	0.004
Java-Bali	0.320	0.312	-0.008
	0.007	0.007	0.010
	0.000	0.000	0.350
Outside Java-Bali	0.275	0.297	0.022
	0.007	0.007	0.010
	0.000	0.000	0.024
Sumatera	0.282	0.291	0.009
	0.007	0.010	0.014
	0.000	0.000	0.509
NTB and NTT	0.248	0.329	0.081
	0.017	0.022	0.028
	0.000	0.000	0.003
Kalimantan	0.284	0.311	0.027
	0.016	0.015	0.022
	0.000	0.000	0.213
Sulawesi	0.288	0.300	0.013
	0.018	0.016	0.024
	0.000	0.000	0.596
Maluku and Papua	0.236	0.283	0.046
	0.016	0.016	0.023
	0.000	0.000	0.042

Source: Authors' analysis based on Susenas 2015-2017 and BPJS-*Kesehatan* administrative data.

Table A6. Distribution of healthcare utilization share across socioeconomic quintiles (2015-2017) (Based on Utilization Rate)

	Hospital Inpatient				Hospital Outpatient				Primary Care Outpatient			
	Inpatient days per 100 individuals in one year	Share (%)	Unit Cost (USD)	Benefit Share % (district unit costs)	Outpatient visit per 100 individuals in a year	Share (%)	Unit Cost (USD)	Benefit Share % (district unit costs)	Outpatient visit per 100 individuals in a year	Share (%)	Unit Cost (USD)	Benefit Share % (district unit costs)
	1	2	3	4	5	6	7	8	9	10	11	12
2015												
Poorest	6.61	11.37	55.29	10.22	NA				NA			
2nd quintile	7.54	12.53	60.85	11.39	NA				NA			
3rd quintile	10.12	17.26	59.36	16.14	NA				NA			
4th quintile	14.03	22.21	63.89	22.11	NA				NA			
Richest	21.95	36.63	63.25	40.14	NA				NA			
Mean	11.50	100.00	60.54	100.00	NA				NA			
2016												
Poorest	5.61	9.54	59.44	8.53	NA				NA			
2nd quintile	7.92	13.63	58.35	12.71	NA				NA			
3rd quintile	9.69	16.46	57.07	15.36	NA				NA			
4th quintile	14.06	23.93	61.14	23.79	NA				NA			
Richest	21.33	36.44	62.42	39.61	NA				NA			
Mean	11.74	100.00	59.91	100.00	NA				NA			
2017												
Poorest	9.72	9.83	38.15	8.82	17.90	8.72	7.98	8.13	194.42	18.95	2.62	17.45
2nd quintile	13.44	13.61	39.65	12.84	28.43	13.27	7.40	12.68	208.03	20.17	2.65	19.20
3rd quintile	16.21	16.78	40.07	15.99	32.23	15.65	7.57	15.01	216.70	21.19	2.69	21.19
4th quintile	21.89	22.92	38.66	22.45	44.38	22.17	7.76	21.85	221.77	21.53	2.77	21.96
Richest	36.01	36.86	38.82	39.90	83.67	40.20	8.12	42.33	193.40	18.16	2.93	20.20
Mean	20.31	100.00	38.91	100.00	43.43	100.01	8.04	100.00	206.94	100.00	2.78	100.00

Figure A1. Total amount of JKN fund disbursed 2014-2017 (trillion IDR)

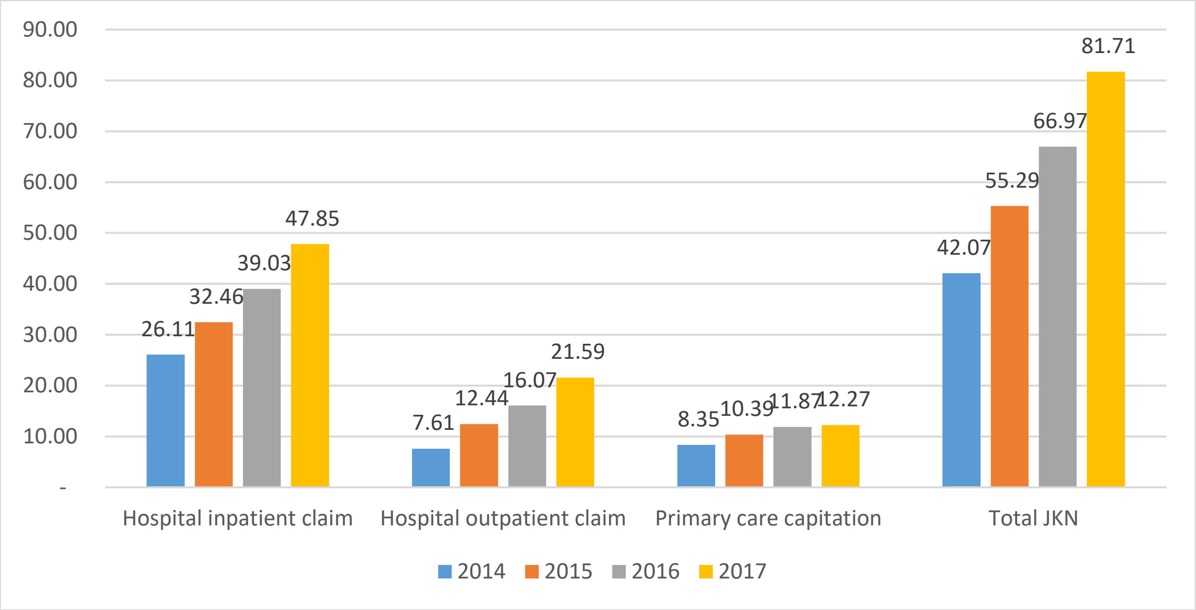

Source: Authors' analysis based on BPJS-*Kesehatan* fund distribution by districts.

Figure A2. Distribution of JKN fund disbursed by type of service 2014-2017

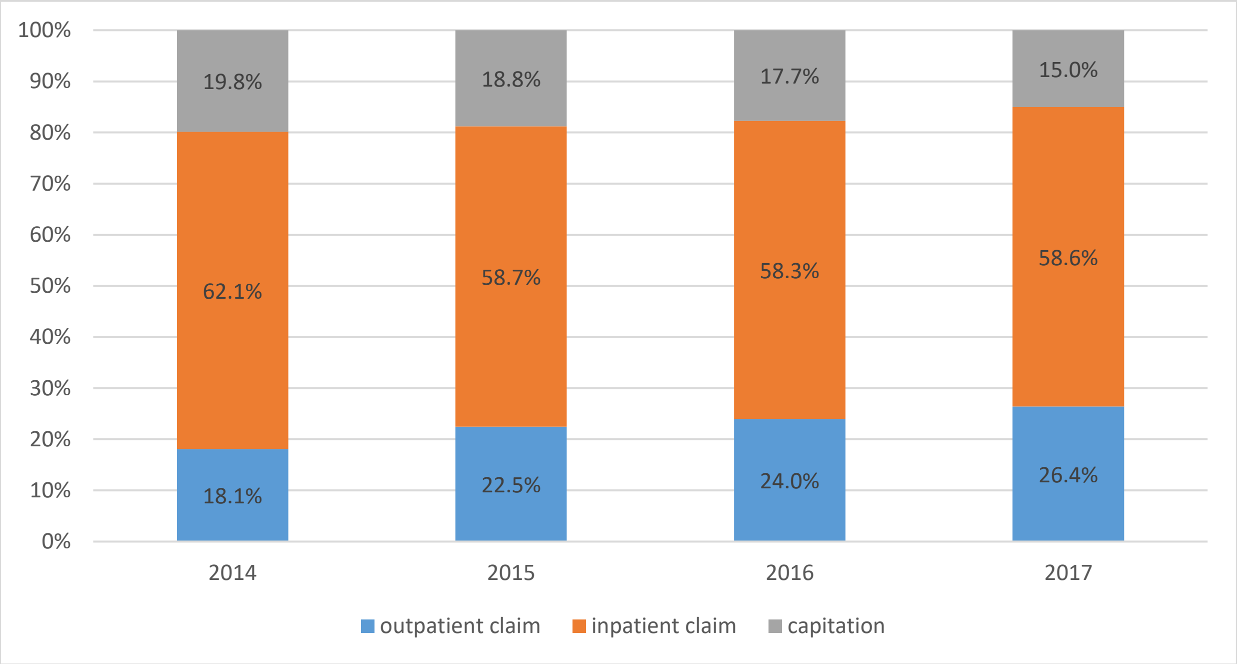

Source: Authors' analysis based on BPJS-*Kesehatan* fund distribution by districts.

Figure A3. Concentration curves for cumulative share of hospital (outpatient and inpatient) and primary care (outpatient) benefit weighted by district unit costs (2015-2017)

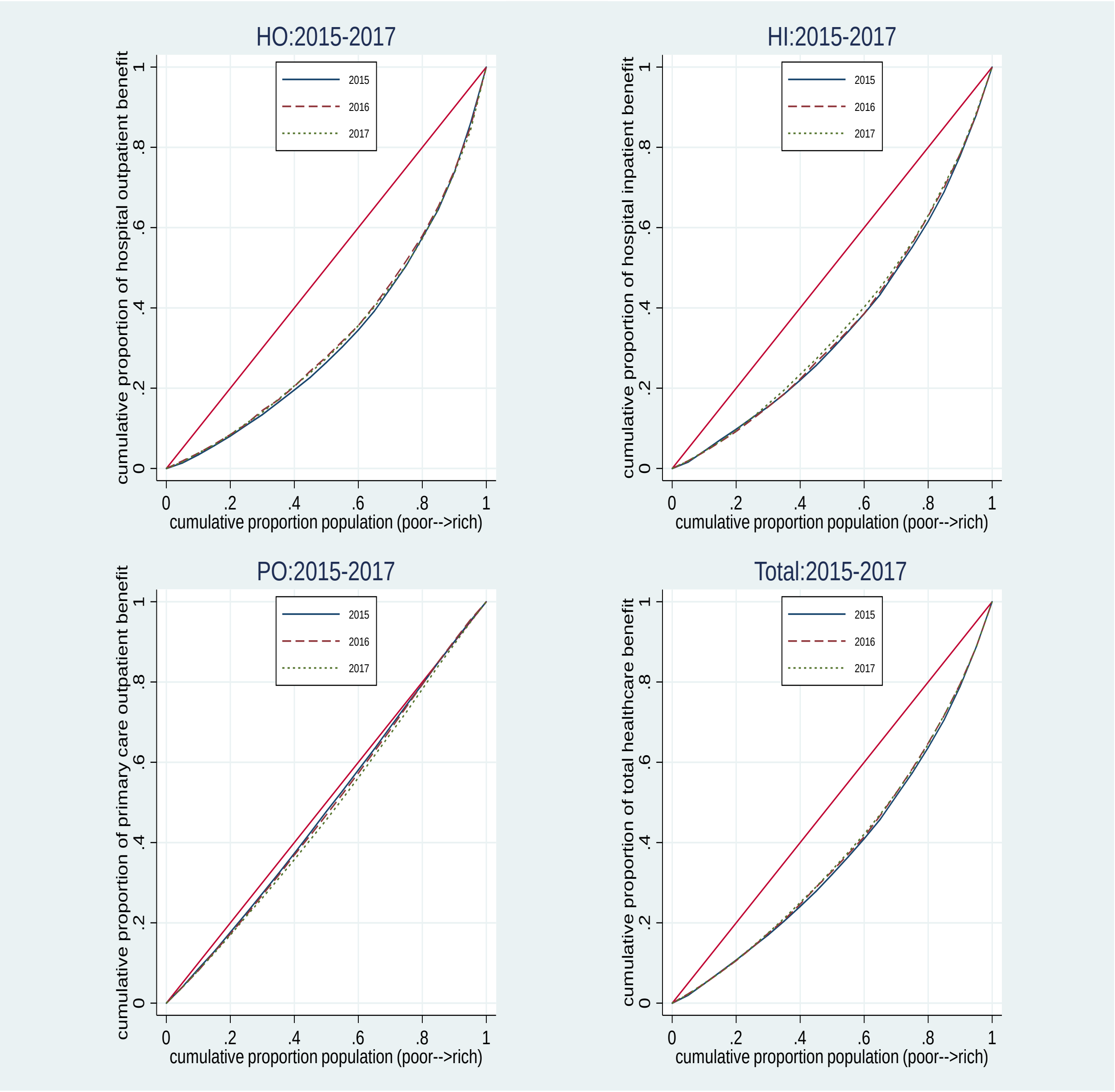

Note: The y-axis plots the cumulative density of healthcare benefits by individuals ranked from the least to highest per capita expenditure per year.

Figure A4. Primary care outpatient (PO) unit cost in district level (2015-2017)

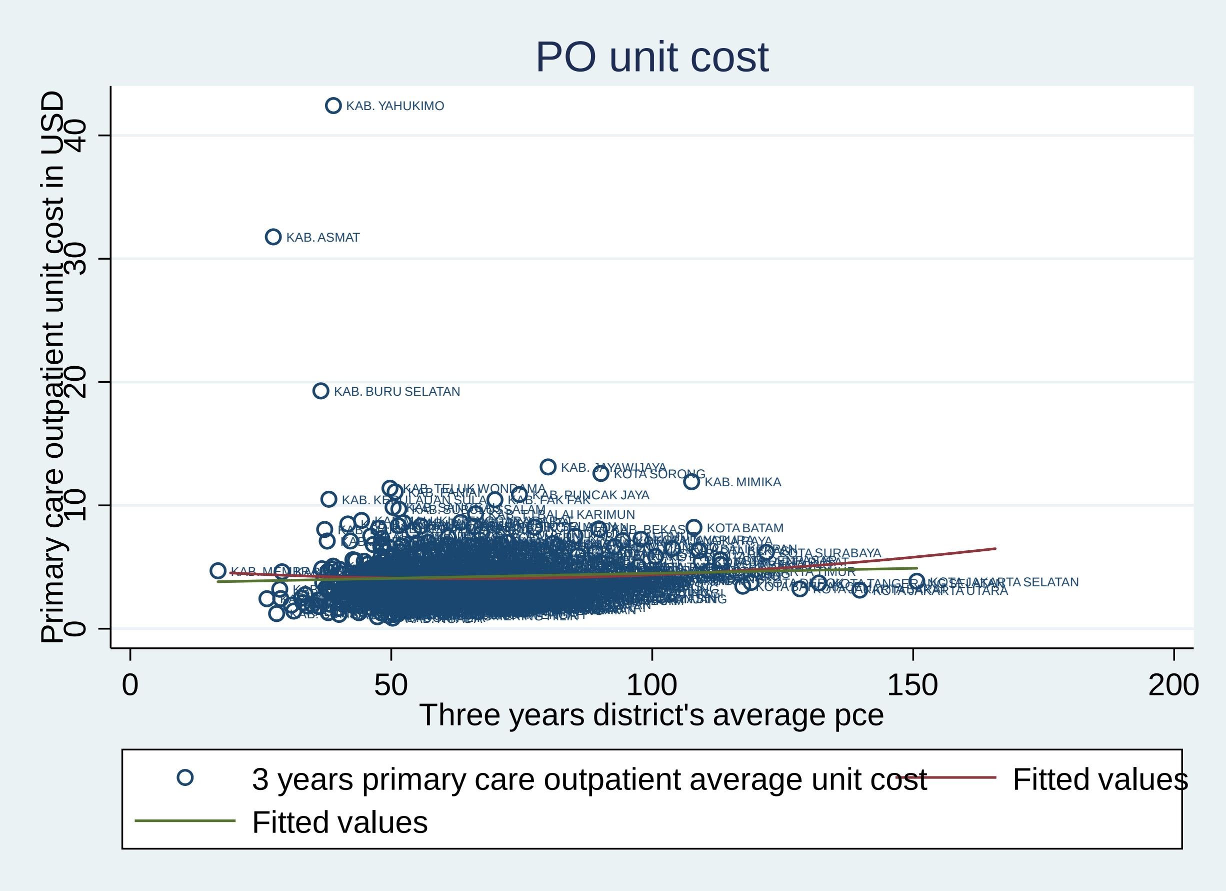

Note: The Y-axis shows three years averages of district specific unit costs derived from the BPJS-Kesehatan records on capitation funds and primary care outpatient contact per district from Susenas. The X-axis shows three years district averages of per capita expenditure derived from Susenas 2015-2017.
